# Coverage of preventive measures and surveillance for neglected tropical diseases in hard-to-reach communities in Ghana

**DOI:** 10.1186/s12889-023-16652-1

**Published:** 2023-09-14

**Authors:** Akua Obeng Forson, Raphael Baffour Awuah, Abdul Rahim Mohammed, Christopher Mfum Owusu-Asenso, Gabriel Akosah-Brempong, Anisa Abdulai, Isaac Kwame Sraku, Shittu B. Dhikrullahi, Sefa Bonsu Atakora, Simon K. Attah, Yaw Asare Afrane

**Affiliations:** 1 Department of Medical Microbiology, Centre for Vector-Borne Diseases Research, University of Ghana Medical School, University of Ghana, Korle-Bu, Accra, Ghana; 2 Department of Medical Laboratory Sciences, School of Biomedical and Allied Health Sciences, University of Ghana, Korle-Bu, Accra, Ghana; 3 Regional Institute for Population Studies (RIPS), University of Ghana, Legon. Accra, Ghana

**Keywords:** Neglected tropical diseases (NTDs), Lymphatic filariasis, Onchocerciasis, Schistosomiasis, Soil transmitted helminths, Ghana

## Abstract

**Background:**

Neglected tropical diseases (NTDs) are a major public health burden which mainly affects poor populations living in tropical environments and hard-to-reach areas. The study sought to examine coverage of preventive efforts, and case surveillance for NTDs in hard-to-reach communities in Ghana.

**Methods:**

The study investigated treatment efforts for lymphatic filariasis (LF), and onchocerciasis and schistosomiasis/soil transmitted helminths (SCH/STH) at household level, in difficult-to-access communities in Ghana. A total of 621 households were sampled from 6 communities in the Western, Oti and Greater Accra regions.

**Results:**

Over 95% of the households surveyed were covered under mass drug administration (MDA) campaigns for lymphatic filariasis (LF) and onchocerciasis. More than 80% of households had received at least two visits by community drug distributors under the MDA campaigns in the last two years preceding the study. In addition, over 90% of households in the LF and onchocerciasis endemic communities had at least one member using anthelminthic medications under the MDA campaigns in the 12 months preceding the study. However, households where no member had taken anthelminthic medications in 12 months preceding the study were over 6 times likely to have someone in the household with LF.

**Conclusions:**

This study determined that SCH/STH, LF and onchocerciasis are of serious public health concern in some communities in Ghana. There is an urgent need for holistic practical disease control plan involving both financial and community support to ensure total control of NTDs in difficult-to-access communities is achieved.

**Supplementary Information:**

The online version contains supplementary material available at 10.1186/s12889-023-16652-1.

## Background

Neglected tropical diseases (NTDs) are a major public health issue affecting poor populations living in tropical environment, and hard-to-reach areas [1, 2]. According to a World Bank study, more than half of sub-Saharan Africa’s population lives on less than US$1.25 per day [3]. This gives an indication of the number of people in the region at risk of NTDs. Physical disabilities, discrimination, and impaired cognitive development resulting from NTDs can also contribute to the social and economic burden of individuals and communities [4, 5]. These interrelated consequences of NTDs further deepen the cycle of poverty as it decreases productivity.

In Ghana, unsafe health practices, limited access to safe water, poor sanitation, and a lack of awareness of the causes of NTDs continue to pose a serious challenge to achieving elimination of NTDs [6, 7]. Nonetheless, there have been efforts to reduce the burden of NTDs in Ghana over the last two decades. For example, the Neglected Tropical Diseases Programme (NTDP) has focused on preventive chemotherapy and transmission control of diseases such as lymphatic filariasis (LF), onchocerciasis, schistosomiasis (SCH), and soil-transmitted helminthiasis (STH) [6–8]. Furthermore, the Ghana NTD master plan has integrated all NTDs, such as LF, SCH, and STH, and included mass drug administration (MDA), and case management based on national strategic priorities [6, 7, 9–11]. In addition, there have been interventions such as the distribution of treated bed nets and indoor residual spraying (IRS) to control malaria in some highly endemic malaria transmission areas, which also happen to be endemic sites for LF [12, 13]. Considering that malaria and LF share the same vectors, these vector control efforts also affect the control and transmission of LF.

Although progress has been made to reduce the burden of selected NTDs in Ghana [7, 14], however, ongoing studies in some districts have found *Anopheles gambiae* mosquitoes infected with the LF parasite (*Wuchereria bancrofti*) in communities where transmission is supposed to have been interrupted [15]. Similarly, *Onchocerca volvulus* in *Simulium damnosum* has been found in some communities where transmission is supposed to have been disrupted [16]. In addition, a major challenge is the fact that some villages endemic for LF, onchocerciasis or both are cut off from the mainland by water, making access to treatment difficult [17]. Studies on SCH and STHs among school children in island communities in Ghana have revealed higher (51–70%) infection rates than those living on the mainland [18]. 

The success of NTD control programs relies on reducing pockets of disease transmission countrywide [19]. As such, preventive measures should be focused on communities at risk of the various NTDs, particularly those that are hard to reach. This is because hard-to-reach communities can be sources of persistent infection that can continue to delay the country’s control and elimination efforts. WHO [1] recommends continued efforts in ensuring treatment of NTDs, as well as improving monitoring and surveillance tools, as important steps in eliminating NTDs.

These reasons underscore the need for studies that examine control efforts in at-risk populations in order to reduce the burden and control NTDs such as LF, onchocerciasis, and SCH/STH in Ghana [10]. This study therefore sought to determine the extent of coverage of preventive measures at the household level for NTDs in hard-to-reach communities in Ghana.

## Methods and materials

### Study design

The study was cross-sectional in design assessing interventions to control LF and onchocerciasis using a household survey among people living in difficult-to-access communities in Ghana.

### Study sites

**Fig. 1:**
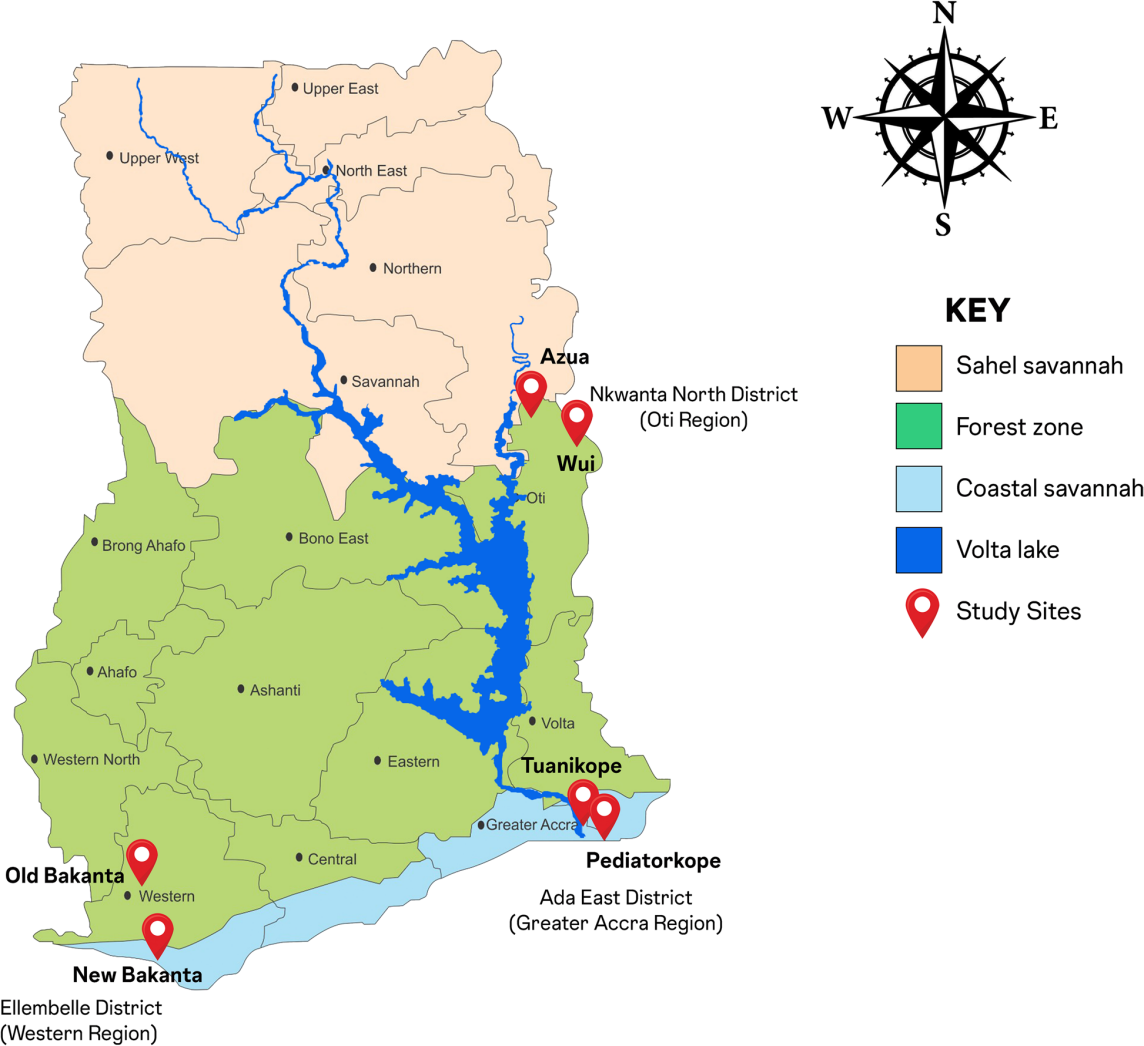
Map showing the study sites used in this study

Data on LF were collected in two communities in the Western Region of Ghana – Old Bakanta and New Bakanta (both in the Ellembelle District). The study sites for onchocerciasis were Azua and Wui in the Nkwanta North District of the Oti Region. Data on SCH and STH were collected in Tuanikope and Pediatorkope in the Ada East District of the Greater Accra Region (Fig. 1). These communities were selected based on their relative risk of NTDs, difficulty in accessing health care for NTDs and other communicable/infectious diseases as well as being hard to reach areas.

### Study procedure

In each community, all households were eligible to be selected to take part in the survey. The total number of households in each community ranged between 60 and 250, depending on the size of the community. A simple random sampling approach was used to select households to participate in the study from May 2019 to September 2019. Estimated sample size was calculated based on the following: margin of error = 5%; design effect = 1; clusters = 1; confidence level = 95%. The combined sample size was 621 households. Table 1 shows the sample sizes and response rate for each study community.

**Table 1:** Number of Respondents recruited into the study per Community

	**Estimated number of households**	**Estimated sample size**	**Response rate % (n)**
Old Bakanta	250	152	88% (133)
New Bakanta	180	123	92% (113)
Azua	300	169	84% (142)
Wui	75	63	91% (57)
Pediatorkope	250	152	89% (134)
Tuanikope	60	53	81% (42)

A household-level questionnaire was administered to heads of households (or the oldest adult household member) in the study communities (see S1 File for Copy of Questionnaire on Socio-Demographic demographic and socio-economic characteristics of the household head, household characteristics, and coverage of preventive measures. Participation was voluntary, and written consent from participants was taken in accordance with the ethical committee’s guidelines.

### Ethical considerations

The study was performed in accordance with the Declaration of Helsinki, and was approved by the Ethics and Protocol Review Committee (EPRC) of the College of Health Sciences, University of Ghana (Ethical No: CHS-ET/M.2–4.10/2018–2019). Written informed consent was provided by all adult participants.

### Data analysis

Univariate results were expressed as percentages (for categorical variables) or means ± standard deviations (for continuous variables). Logistic regression analysis was used to assess the association between preventive measures and the prevalence of LF and onchocerciasis. The data were analysed with SPSS (version 23).

## Results

### Background characteristics of household heads

Table 2 shows the socio-demographic characteristics of household heads for the overall sample, and by study community. The mean age of household heads in the overall sample was 43.6 years. In all the study communities, there were more male headed households (65%) than female headed households (35%). A majority of household heads in the overall sample had some formal education (58%), were Christian (64%), belonged to a non-Akan ethnic group (61%) and were farmers (40%).

**Table 2:** Characteristics of Household Heads

**Characteristic**	**Total** **(** ***n*** ** = 621)**	**Old Bakanta** **(** ***n*** ** = 133)**	**New Bakanta** **(** ***n*** ** = 113)**	**Azua** **(** ***n*** ** = 142)**	**Wui** **(** ***n*** ** = 57)**	**Pediator-kope** **(** ***n*** ** = 134)**	**Tuani-kope** **(** ***n*** ** = 42)**
**Age (years)**	43.6 (± 16.8)	44.5 (± 16.5)	45.4 (± 16.5)	39.7 (± 16.4)	43.6 (± 16.8)	40.1 (± 12.9)	49.0 (± 17.7)
**Sex**							
Male	405 (65.2)	71 (53.4)	54 (47.8)	118 (83.1)	46 (80.7)	84 (62.7)	32 (76.2)
Female	216 (34.8)	62 (46.6)	59 (52.2)	24 (16.9)	11 (19.3)	50 (37.3)	10 (23.8)
**Education**							
No education	260 (41.9)	45 (33.8)	34 (30.1)	105 (73.9)	45 (78.9)	28 (20.9)	3 (7.1)
Primary education	95 (15.3)	16(12.0)	14 (12.4)	11 (7.7)	4 (7.0)	34 (25.4)	16 (38.1)
Lower secondary	195 (31.4)	51 (38.3)	44 (38.9)	17 (12.0)	4 (7.0)	59 (44.0)	20 (47.6)
Higher secondary	44 (7.1)	15 (11.3)	9 (8.0)	7 (4.9)	3 (5.3)	8 (6.0)	2 (4.8)
Tertiary	27 (4.3)	6 (4.5)	12 (10.6)	2 (1.4)	1 (1.8)	5 (3.7)	1 (2.4)
**Religion**							
No religion	20 (3.2)	2 (1.5)	0 (0)	1 (0.7)	5 (8.8)	11 (8.2)	1 (2.4)
Christian	400 (64.4)	128 (96.2)	110 (97.3)	28 (19.7)	18 (31.6)	96 (71.6)	20 (47.6)
Muslim	27 (4.3)	3 (2.3)	1 (0.9)	12 (8.5)	0 (0)	11 (8.2)	0 (0)
Traditionalist	172 (27.7)	0 (0)	0 (0)	101 (71.1)	34 (59.6)	16 (11.9)	21 (50.0)
Other religion	2 (0.3)	0 (0)	2 (1.8)	0 (0)	0 (0)	0 (0)	0 (0)
**Ethnicity**							
Akan	244 (39.3)	132 (99.2)	112 (99.1)	0 (0)	0 (0)	0 (0)	0 (0)
Ga-Dangbe	131 (21.1)	0 (0)	1 (0.9)	4 (2.8)	1 (1.8)	129 (96.3)	0 (0)
Ewe	51 (8.2)	0 (0)	0 (0)	138 (97.2)	56 (98.2)	5 (3.7)	42 (100)
Other (mixed ethnicity)	195 (31.4)	1 (0.8)	0 (0)	0 (0)	0 (0)	0 (0)	0 (0)
**Occupation**							
No occupation	57 (9.2)	31 (23.3)	6 (5.3)	1 (0.7)	3 (5.3)	15 (11.2)	1 (2.4)
Farmer	250 (40.3)	12 (9.0)	27 (23.9)	132 (93)	51 (89.5)	21 (15.7)	7 (16.7)
Sales/trading	145 (23.3)	43 (32.3)	34 (30.1)	6 (4.2)	3 (5.3)	52 (38.8)	7 (16.7)
Professional/tech	112 (27.2)	47 (35.3)	46 (40.7)	3 (2.1)	0 (0)	46 (34.3)	27 (64.3)

Values are n (± SD) or n (%)

### Household characteristics assessed in the study sites

The median monthly household income ranged from GHC150 (≈$27) in Azua, to GHC350 (≈$63) in Wui. The average household size was 5.8, although in the community of Azua, the average household size was higher (7.3) (Table 3).

**Table 3:** Selected Household Characteristics

Characteristic	**Total**	**Old Bakanta**	**New Bakanta**	**Azua**	**Wui**	**Pediatorkope**	**Tuanikope**
	(*n* = 621)	(*n* = 133)	(*n* = 113)	(*n* = 142)	(*n* = 57)	(*n* = 134)	(*n* = 42)
**Median monthly household income (GHC)**	200	200	300	150	350	200	300
**Average number of household members**	5.8 (± 3.5)	5.2 (± 3.0)	5.7 (± 3.0)	7.3 (± 4.8)	6.2 (± 3.2)	5.2 (± 2.6)	4.9 (± 2.6)
**Availability of toilet facility**							
Yes (private or public)	292 (47.0)	94 (70.7)	94 (83.2)	8 (5.6)	45 (78.9)	18 (13.4)	33 (78.6)
No (open defaecation)	329 (53.0)	39 (29.3)	19 (16.8)	134 (94.4)	12 (21.1)	116 (86.6)	9 (21.4)
**Household water source**							
Protected	282 (45.4)	86 (64.7)	61 (54.0)	103 (72.5)	19 (33.3)	11 (8.2)	2 (4.8)
Unprotected	339 (54.6)	47 (35.3)	52 (46.0)	39 (27.5)	38 (66.7)	123 (91.8)	40 (95.2)
**Presence of animals in dwelling**							
Pets/livestock/fowls	489 (78.7)	101 (75.9)	85 (75.2)	122 (85.9)	48 (84.2)	99 (73.9)	34 (81.0)
No animals	132 (21.3)	32 (24.1)	28 (24.8)	20 (14.1)	9 (15.8)	35 (26.1)	8 (19.0)

Values are n (± SD) or n (%)

Regarding water and sanitation, more than half (55%) of the respondents reported that water for the household use was from unprotected sources (such as surface water and borehole). All other household heads (45%) reported that water for their household use was from protected sources (such as pipe in the house or public tap/standpipe). More than half of household heads (53%) reported lack of a toilet facility, and indicated that members of the household as a result practiced open defecation.

### Coverage of preventive measures

Approximately 96% and 98% of households in the LF and onchocerciasis endemic communities were covered under MDA campaigns (Table 4). Furthermore, 82% and 86% of households in the LF and onchocerciasis endemic communities respectively had received at least two visits by community drug distributors (CDDs) under the MDA campaigns in the last two years preceding the study.

**Table 4:** NTD control strategies

	**LF endemic communities** ***n*** ** = 246 (%)**	**Onchocerciasis endemic communities** ***n*** ** = 199(%)**	**SCH/STH endemic communities** ***n*** ** = 176 (%)**
**Household covered in MDA**			
Yes	236 (95.9)	194 (97.5)	0 (0)
No	10 (4.1)	5 (2.5)	176 (100)
**Frequency of MDA**			
None	22 (8.9)	9 (4.5)	176 (100)
Once	21 (8.5)	19 (9.5)	-
Twice or more	202 (82.1)	171 (86.0)	-
**Use of anthelminthic medications**			
Yes	222 (90.2)	188 (94.5)	8 (4.5)
No	24 (9.8)	11 (5.5)	168 (95.5)
**Use of bed nets** ^**a**^			
Never/hardly ever	52 (21.1)	-	-
Often	194 (78.9)	-	-

*N* Total number, *%* Percentage

^a^Use of bed nets was not assessed in onchocerciasis and SCH/STH endemic communities

In addition, 90% and 95% of households in the LF, and onchocerciasis endemic communities had at least one member using anthelminthic medications under the MDA campaigns in the 12 months preceding the study (Table 4). In the LF endemic communities, 79% of households often used treated bed nets to protect themselves against mosquitoes that transmit the infection. In the SCH/STH endemic communities there had been no MDA visits in the last two years preceding the study, and only 5% of households self-administered anthelminthic medications in the 12 months preceding the study.

### Association between interventions and disease burden

Table 5 shows the logistic regression analyses assessing the association between control strategies and the prevalence of the NTDs. Coverage, and frequency of MDA campaigns were not included in the analysis due to bias estimates or non-assessment of those variables in some study communities. For example, frequency of MDA was not assessed in the SCH/STH endemic communities since no household in the study communities were covered under MDA campaigns.

**Table 5:** Association between preventive measures and the prevalence of NTDs in households

	**LF endemic communities** **OR (95% CI)**	**Onchocerciasis endemic communities** **OR (95% CI)**	**SCH/STH endemic communities** **OR (95% CI)**
**Use of bed nets** ^**a**^			
Never/hardly ever	1.00	-	-
Often	2.63 (0.30–22.81)	-	-
**Use of anthelmintic drugs**			
Yes (RC)	1.00	1.00	1.00
No	6.17 (1.38–27.66) ^+^	1.04 (0.26–4.06)	0.95 (0.11–8.27)

*OR* Odds ratio, *95% CI* 95% Confidence Interval, *RC* Reference category

^+^*p* > 0.05. Analyses adjusted for household monthly median income, household size and household water source. Coverage and frequency of MDAs were not included in the analysis for LF, onchocerciasis and SCH/STH due to bias estimates

^a^Use of bed nets was not included in the analysis for onchocerciasis and SCH/STH due to non-assessment in the study communities

Adjusted odds ratio analyses revealed that households where no member had taken anthelminthic medications in the 12 months preceding the study were over 6 times more likely to have someone in the household with LF

## Discussion

Lymphatic filariasis (LF), onchocerciasis, schistosomiasis, and soil transmitted helminths are important neglected tropical diseases in sub-Saharan Africa, including Ghana. This study investigated treatment efforts for lymphatic filariasis (LF) and onchocerciasis at the household level in difficult-to-access communities in Ghana. Findings from this study revealed households where no member had taken anthelminthic medications in the 12 months preceding the study were over 6 times likely to have someone in the household with LF, and self-reported household prevalence for onchocerciasis was higher compared to LF and SCH/STH. This finding corroborates the assertion that even though a combination of effective vector control and access to treatment will reduce the prevalence of LF in communities, MDA is a more effective control strategy [20]. This study has revealed there is a need to assess the current control interventions on onchocerciasis in Ghana in order to ensure the prevalence of onchocerciasis is reduced to a level where it ceases to be a public health concern.

This study revealed that the majority of households covered under preventive treatment for LF and onchocerciasis had received at least two MDA visits in the last two years preceding the study and had at least one household member using anthelminthic drugs within the last year preceding the survey. On the contrary, there had been no MDA visits in the SCH/STH endemic communities in the last two years preceding the survey, and only 5% of households reported using anthelminthic drugs in the last year. These findings suggest that control strategies for NTDs have been unequal in difficult-to-access communities in Ghana. In addition, it has been suggested that to help control NTDs, there should be sustained, expanded, and extended drug access programs to ensure the necessary supply of drugs, as well as addressing identified drawbacks to improve the effectiveness of other interventions [21]. Studies conducted in Côte d’Ivoire by Loukouri et al. [20] have revealed that annual and semi-annual mass drug administration for Lymphatic Filariasis and Onchocerciasis helped reduced the prevalence of infection.

However, taking into account the logistical challenges associated with MDA and other vector control interventions, achieving coverage of the entire population is hardly ever achieved, particularly in difficult-to-access communities, and this results in residual infections [22]. Therefore, it is essential that the entire coverage of these interventions be ensured by district health authorities to achieve control and elimination of LF and onchocerciasis with time. There is evidence to show that knowledge of a disease condition influences attitude and practice, which improves compliance with treatment and subsequently leads to a reduction in prevalence [23]. Periodic health education, facilitated by well-trained community drug distributors, and focusing on gaps in knowledge, should be pursued in the study communities.

The implementation of semi-annual community-wide MDA in the SCH/STH endemic study communities should be vigorously pursued and sustained. Evidence from previous studies in similar settings show that MDA programs have been largely effective against SCH and STH particularly among school-age children [24, 25].

## Conclusions

Optimizing surveillance, and improving the success rates of the MDA and other vector control programs in Ghana depends in large part on visits facilitated by CDDs. The findings suggest that CDDs in communities where NTDs are endemic should be given well-defined mandates, regularly trained, and supervised, and given the necessary resources to execute their mandate considering the relative precarious circumstances in the communities they serve. Over time, this will guarantee the success of strategies to control and possibly eliminate NTDs in difficult-to-access communities.

### Supplementary Information

**Additional file 1: S1.** File Household Questionnaire-2.

## Data Availability

The datasets used and/or analysed during the current study are available from the corresponding author on reasonable request.

## Abbreviations

NTDs: Neglected tropical diseases
SCH: Schistosomiasis
STH: Soil transmitted helminths
LF: Lymphatic Filariasis
GHC: Ghana cedis
