# Supplementary material for: Coverage of preventive measures and surveillance for neglected tropical diseases in hard-to-reach communities in Ghana
Source: BMC Public Health. 2023 Sep 14;23:1784. doi: 10.1186/s12889-023-16652-1 (PMC10500849; doi:10.1186/s12889-023-16652-1)
Supplement: Supplementary file 1 — Additional file 1: S1. File Household Questionnaire-2. [file 12889_2023_16652_MOESM1_ESM.docx]

**Household Questionnaire**

**Interviewer Name:** ________________________

**Respondent ID:** __________________________

**Date of interview:** ________________________ (dd/mm/yyyy)

**Community:** _____________________________

**District: _________________________________**

**Demographic and socio-economic characteristics**

**Q1.** Sex of household head.

1 = Male 2 = Female

**Q2.** How old are you? (estimated age in years) ___________

**Q3.** Ethnic group of household head.

1 = Akan 2 = Ga-Dangbe 3 = Ewe 4 = Ga 5 = Hausa

6 = Other (specify) _____________________

**Q4.** Religious affiliation of household head.

1=No religion 2=Christian 3=Muslim 4=Traditional/spiritualist

5=Other (Specify) _____________________

**Q5.** Marital status of household head.

1 = Not married 2 = Married 3 = Divorced/separated 4 = Widow/widower

**Q6.** What is the highest level of formal education you have attained or completed?

1 = No education 2 = Primary 3 = Middle/Junior High 4 = Senior High

5 = Tertiary

**Q7.** Occupation of household head.
 1 = No occupation 2 = Farmer 3 = Professional/technical

4 = Sales/Trading 5 = Unskilled manual 6 = Other (specify)________________

**Q8.** Average monthly household income? GHC___________

**Household Characteristics**

‘

**Q9.** How many people live in your household? _____ people

**Q10.** What kind of toilet facility do members of your household usually use?

1 = WC within 2 = Pit latrine within 3 = Public toilet 4 = Free range

5 = Other__________________

**Q11.** What is the main source of water for household uses/chores?

1 = Pipe in the house 2 = Public tap/standpipe 3 = Tube well or borehole

4 = Dug well 5 = Water from spring 6 = Rainwater

7 = Tanker truck 8 = Cart with small tank 9 = Sachet water

10 = Surface water (river, pond etc.) 11 = bottled water 12 = Other___________

**Q12.** Main material of exterior walls of household dwelling

1 = Cement 2 = Bricks 3= Wood (plywood) 4 = Bamboo

5 = Mud 7 = Other _______________

**Q13.** Main material of roofing of household dwelling

1 = Roofing sheets 2 = Wood 3= Metal 4 = Thatched roof 5 = Other ____________

**Q14.** Main material of the floor of household dwelling

1 = Cement 2 = Carpet 3= Wood 4 = Tiles

5 = Mud 7 = Other ___________________

**Q15.** Number of windows in household dwelling? ________ windows

**Q16.** What kind of animals (including livestock) do you have in this household?

1 = Pets (eg. dog, cat) 2 = Livestock (eg. cattle, sheep, goat)

3 = Fowl (eg. chicken, guinea fowl) 4 = Other______________________

**Knowledge and Perception on causes of NTDs**

**Q17.** Have you ever heard of lymphatic filariasis? (Give a basic/lay explanation of the disease)

1 = Yes 2 = No

**Q18.** Do you know the causes/transmission of LF?

1 = Yes 2 = No

If yes, list them____________________________________

**Q19.** Have you ever heard of onchocerciasis? (Give a basic/lay explanation of the disease)

1 = Yes 2 = No

**Q20.** Do you know the causes/transmission of onchocerciasis?

1 = Yes 2 = No

If yes, list them____________________________________

**Q21.** Have you ever heard of schistosomiasis? (Give a basic/lay explanation of the disease)

1 = Yes 2 = No

**Q22.** Do you know the causes/transmission of schistosomiasis?

1 = Yes 2 = No

If yes, list them____________________________________

**Q23.** Have you ever heard of soil-transmitted helminths? (Give a basic/lay explanation of the disease)

1 = Yes 2 = No

**Q24.** Do you know the causes/transmission of soil-transmitted helminths?

1 = Yes 2 = No

If yes, list them____________________________________

**Control of NTDs**

**Q25**. Is your household covered in the preventive treatment for NTD?

1 = Yes 2 = No

**Q26.** How many times in the last 2 years have you received the mass drug administration (MDA) programme visits?

1 = None 2 = Once 3 = Twice 4 = Three times 5 = Four or more

**Q27.** How often do household members use treated bed nets?

1 = Never 2 = Hardly ever 3 = Often 4 = Always

**Q28.** Has anyone in your household taken anthelmintic drugs within the **last one year**?

1 = Yes 2 = No

If yes, name of drug_____________________ and where did you get it from _____________

**Q29.** If yes, do household members **often** adhere to the dosage regime of the drug?

1 = No 2 = Sometimes 3=All the time

If **no,** why?

1=Adverse effects 2= Forgetfulness 3= Difficulty in swallowing

4. Other, mention____________________________

**Q30.** If adverse effects, indicate _________________________________

­­­­­­­­­­­­­­­­­_________________________________

_________________________________

**Q31.** Do members of your household sometimes share their drugs?
 1 = Yes 2 = No

**Q32.** Has your household ever been covered under the indoor residual spraying (IRS) programme?

1 = Yes 2 = No

**Q33.** Do you know your community drug distributor?

1 = Yes 2 = No

**Burden of NTDs**

**Burden of NTDs**

**Q34.** Has anyone in your household exhibited any signs of *LF* in the last one year?

1 = Yes 2 = No

**Q35.** If yes, how many household members have had *LF* in the last one year? ______ household members

**Q36.** Has anyone in your household had *onchocerciasis* in the last one year?

1 = Yes 2 = No

**Q37.** If yes, how many household members have had *onchocerciasis* in the last one year? ______ household members

**Q38.** Has anyone in your household had *schistosomiasis* in the last one year?

1 = Yes 2 = No

**Q39.** If yes, how many household members have had *schistosomiasis* in the last one year? ______ household members

**Q40.** Has anyone in your household had *soil-transmitted helminths* in the last one year?

1 = Yes 2 = No

**Q41.** If yes, how many household members have had *soil-transmitted helminths* in the last one year? ______ household members.
